# How on‐demand agency of anonymous group exercise membership supports emergence‐based social identity transition in mid‐life

**DOI:** 10.1111/bjso.70022

**Published:** 2025-11-14

**Authors:** Toby Richards, Matthew J. Easterbrook, Matthew J. Slater, Melissa Day, Sean G. Figgins

**Affiliations:** ^1^ School of Psychology University of Sussex Brighton UK; ^2^ School of Health, Education, Policing, and Sciences Staffordshire University Stoke‐on‐Trent UK; ^3^ Institute of Applied Sciences University of Chichester Chichester UK

**Keywords:** anonymity, midlife, online exercise groups, social identity, social identity deconstruction, social identity model of identity change (SIMIC)

## Abstract

Midlife's challenges, changes and demands can create barriers to maintaining group activities, which, for some, include attending in‐person group exercise classes. As a potential solution, on‐demand group exercise platforms offer agency over participation, anonymity and community interaction. This research explores how social identification processes shape participation within an on‐demand group exercise platform. Twenty on‐demand group exercise participants aged 40–64 were recruited for three data collection stages: (1) an initial semi‐structured interview on exercise history and on‐demand usage; (2) a two‐week post‐exercise diary capturing social identification experiences and (3) a follow‐up interview to discuss topics from the first two stages. Results highlight how, through anonymous participation in on‐demand group exercise, participants experienced a sense of agency, inclusion and community while feeling socially supported both during and after participation. Findings from this study suggest four factors that can impact social identification within on‐demand exercise platforms, namely, (a) creating a collective learning event to foster unity, (b) providing anonymity and agency to enable increased exercise trial, (c) enabling exercise participation from self‐excluded groups and (d) amplifying life‐stage similarity and support both on‐screen and via social media.

## INTRODUCTION

Midlife is a pivotal period of transformational life change, bridging an individual's transition between young adulthood and older age. As a point of intergenerational connection, those in midlife often have responsibilities for individuals both younger and older than themselves across multiple contexts, including personally (e.g., at home), professionally (e.g., in the workplace) and culturally (e.g., broader society). Collectively, this period brings personal and social changes with challenges (e.g., declining physical capabilities, mental health risks), demands (e.g., caregiving, career, financial responsibilities) and opportunities (e.g., career peaks, established relationships) unique to this life stage (Infurna et al., 2020; Lachman et al., 2015). These changes can contribute to a phase of self‐reflection and life revaluation (Komarovsky, 1986), and sudden changes in careers and personal lives (Martin & Prosen, 1972), often referred to as a midlife crisis (Jaques, 1993). Research suggests that midlife demands affect social, physical and mental health (Arnett, 2018; Gomez‐Bernal et al., 2019). One of the major reasons for this impact is that the demands of midlife reduce time for adults to engage in health‐supporting activities such as physical activity.

Decades of research demonstrate that physical activity supports both physical and mental health (Powell et al., 2011). In midlife, physical activity improves heart health, reduces fatigue, (Čolović et al., 2020; Debi et al., 2019), supports brain health and may lower the risk of Alzheimer's and dementia (Ai et al., 2024; Chang et al., 2010; Hörder et al., 2018), while helping to slow physical decline (Gabriel et al., 2017). In contrast, inactivity is linked to chronic illnesses and psychological issues like depression and anxiety (Cunningham et al., 2020). Long‐term data show that being active in midlife increases the likelihood of staying active in later life (Aggio et al., 2017), and yet, inactivity tends to increase from midlife onward in both sexes (Ekkekakis et al., 2011; NHS, 2017). Therefore, encouraging midlife adults to exercise benefits individuals (e.g., improved quality of life), society (e.g., reduced health care burden) and the economy (e.g., sustained employment). Therefore, understanding current barriers to exercise is essential, with research indicating practical (e.g., cost, access), safety (e.g., injury risk) and psychological factors (e.g., low motivation) as reasons for reduced engagement in exercise (Spiteri et al., 2019). To overcome these barriers, many midlife adults have turned to home‐based, online exercise because it is more convenient (i.e., fits in with the increased demands of midlife) and poses fewer safety risks than more traditional forms of in‐person exercise (Subramaniam et al., 2022). However, given that social factors have been shown to be central to exercise engagement (Branch et al., 2024; Stevens et al., 2017) research is needed to explore the role of social processes in online exercise contexts where participation can come with varying levels of interaction with others (e.g., you can exercise asynchronously alone or synchronously with other participants and instructors).

Research into online exercise is currently limited. However, some notable studies exist. A study using content analysis of journals, articles, books and newspapers suggested that participation in online platforms such as Peloton and Zwift can facilitate behaviour change, improve well‐being and help sustain physical activity (Supriyanto & Liu, 2021). Additionally, a study by Davies et al. (2024) of 663 Peloton users found that a strong sense of community influenced participants' motivation to exercise and intent to maintain consistent participation on the platform. Peloton users who felt a community connection engaged more on social media and used their equipment more often. This suggests that social dynamics influence the quality of motivation and sustained involvement in online exercise groups. Consequently, the primary aim of our research was to explore the role of social dynamics in online exercise contexts. We did so, specifically, by using the social identity approach as a lens to explore midlife adults' lived experience of participation in one asynchronous online exercise context.

### The social identity approach

Given that midlife adults are going through a transitional stage which is impacting their ability to exercise, the social identity approach (which incorporates social identity and self‐categorization theories; Tajfel et al., 1979; Turner et al., 1987), provides a useful framework for understanding exercise behaviours of adults during their midlife transition. Social identity theory suggests that when individuals align with a group, they embrace that group's behaviours, norms and values (Tajfel et al., 1979). Additionally, self‐categorization theory indicates that people classify themselves and others into social groups, which affects their behaviour, perceptions of and sense of belonging to a particular social category (Turner et al., 1987). Essentially, individuals perceive themselves as ‘we’ and ‘us’ rather than ‘I’ and ‘me’, promoting collective behaviours and supporting the adoption of shared group norms. Research underpinned by the social identity approach suggests that group memberships (e.g., via the Social Identity Model of Identity Change; SIMIC) and group identification impact life‐stage changes and intentions to exercise (Haslam et al., 2019; Rowe & Slater, 2021). Given the interaction between the life stage (i.e., the midlife transition) and exercise behaviour—whereby the demands of midlife impact on people's ability to engage in exercise—this study used SIMIC and social identity theory as lenses to explore midlife adults' experiences of participating in an asynchronous on‐demand group exercise platform.

### The social identity approach to life stage transitions

Midlife represents a life stage transition that contains inherent uncertainty (Aldwin & Levenson, 2001). SIMIC explains how group memberships influence individual responses to significant life events (e.g., retirement) and their effects on health and well‐being (Haslam et al., 2023). The premise of SIMIC is that life events can be perceived as stressful and thus compromise well‐being when they lead to identity loss (Praharso et al., 2017). A loss of group membership, along with the associated social and psychological resources, has been demonstrated to affect people's sense of meaning in life and diminish their perceived control over their lives due to this change (Haslam, Haslam, et al., 2021). The SIMIC model suggests that preserving identity continuity or developing new group memberships can alleviate the negative impacts of life changes, build resilience and enhance well‐being during transitions (Haslam, Lam, et al., 2021).

Given that midlife often results in increased demands on time (e.g., children, career, caring responsibilities) and physical challenges (e.g., injury, reduced mobility and physical capabilities) this can lead to reduced or even discontinued engagement in exercise which, subsequently, leads to the loss of valued social identities (e.g., as an exerciser and/or as a member of a specific exercise group). As many midlife adults are turning to online exercise to fill this void, exploring the role such contexts play in this transition is one focus of the present research (RQ1). That is, whether and how online exercise contexts provide participants with a sense of group membership may mitigate the (in‐person) exercise identity loss resulting from the challenges and demands of the midlife transition.

### The social identity approach to exercise

The social identity approach has also been applied to exercise settings, with evidence showing that people are drawn to and remain committed to exercise groups that reflect their self‐concept (Stevens et al., 2017, 2018). A growing body of research has demonstrated the importance of group identification for in‐person exercise. For example, group identification is associated with increased effort (Stevens et al., 2019), improved attendance (Stevens et al., 2022a) and a higher likelihood of continued participation (Rowe & Slater, 2021). Though there is less research exploring social identity in online contexts, some initial research has explored social identity in online exercise contexts. For example, Stevens et al. (2022a) found that instructor communication enhanced workout performance, particularly among participants with strong group identification. Similarly, a randomized controlled trial by Beauchamp et al. (2021) demonstrated that an online group exercise programme emphasizing shared identity improved both the mental and physical health of older adults (aged 65+). While understanding of the health and motivational benefits of social identity in online exercise is growing, the factors that facilitate social identification in online exercise contexts remain underexplored. Research conducted in other online contexts (e.g., exploring social identification processes on China's largest online discussion forum) suggests that social media can enhance social identity and group membership through interaction and knowledge exchange (Cheng & Guo, 2015), fostering trust and empathy (Zhao et al., 2020). In an initial study exploring social identity in online exercise, Richards et al. (2025) found that midlife Zwift users' participation was shaped by social identification with other riders driven by shared age and life stage, in‐ride text chatting, avatar‐based cycling and post‐ride engagement via dedicated social media groups (Richards et al., 2025).

Despite these promising initial findings, unlike Zwift, other online exercise contexts exist where participation is asynchronous and does not allow for real‐time interaction. While this provides anonymity that may hold some benefits for participants—e.g., somebody exercising at home without others to compare themselves against might feel more confident to try and stick with new exercise—it does lack real‐time interaction proposed as central to fostering social identification. Some research in other online contexts offers conflicting evidence on the impact of anonymity on social identification.

In an experimental study examining how anonymity in online group communication influences how people follow and adopt group norms or behaviours, especially when those norms are subtly introduced or suggested, Postmes et al. (2001) discovered that anonymity reinforced group norms by hiding individual traits, shifting focus from ‘who I am’ to ‘who we are’, thereby strengthening group identity and encouraging prosocial behaviour. Conversely, Kim et al. (2019) argued that anonymity weakens group identification by fostering detachment for participants in an online discussion‐based community in South Korea. Thus, given these mixed findings, more research is needed to unpack how and whether anonymity impacts social identification in online, asynchronous groups. Considering this in relation to online exercise, as on‐demand exercise platforms grow in popularity, it remains unclear whether users can feel a sense of group identification. To date, however, there are no studies on social identification within completely anonymous, asynchronous group exercise contexts. Consequently, another aim of this research was to examine social identification processes (e.g., what facilitates social identification) within online group exercise environments where people participate asynchronously and without the ability to interact with others in real time (RQ2). Specifically, we sought to explore if participants in these groups felt a sense of group identification in these contexts and, if so, what facilitated this identification.

### The present study

The above illustrates how on‐demand group exercise creates a unique context for exploring identity processes. Features like anonymity, asynchronous participation and on‐demand access change the dynamics of identification compared to in‐person or live‐streamed group classes. This study focused on a popular online fitness platform that offers pre‐recorded group workouts, accessible anytime and anywhere. Users can follow choreographed classes in various disciplines, including dance, yoga, weights, cardio and meditation, with differing intensities (e.g., low to high) and durations (e.g., 15 to 60 minutes). Classes vary from equipment‐free to equipment‐based options and are filmed in diverse locations, featuring different instructor styles and participant viewing angles. While users engage in workouts anonymously, the platform encourages class engagement via content launches and specialized social media groups. Using interviews, this study explored on‐demand participation among midlife adults through a social identity lens and had two aims: (1) to explore the role that on‐demand group exercise participation played in participants' midlife transition and (2) to understand the facilitators of social identification related to this activity.

## METHOD

### Philosophical underpinning

This research is grounded in ontological relativism (i.e., the belief in multiple realities) and epistemological constructivism (i.e., the view that knowledge is subjective and socially constructed). Ontological relativism is well suited to the study's aims of exploring individual experiences of group identity formation, recognizing that participants' realities are constructed and dependent on context. Epistemological constructivism forms the foundation for exploring how group meanings, norms and a sense of belonging develop through participation and interaction. The study combined reflective methods (i.e., interviews; Smith & Sparkes, 2016) with proximal ones (i.e., diaries; Day, 2016) to develop an in‐depth understanding of participants' experiences before (interview 1), as near‐during as possible (post‐exercise diary) and after (interview 2) on‐demand participation. The research questions (i.e., exploring experiences of social identification and sources of group belonging among midlife adults using on‐demand exercise) required a stance that viewed identity as dynamic and relational, not fixed or objectively determined. Therefore, adopting a relativist‐constructivist approach enabled the research to reveal nuanced, evolving identity processes.

### Participants

Purposeful sampling recruited 20 middle‐aged adults (40–64) who actively participated in and identified with a popular on‐demand group exercise platform. Participants were recruited via social media posts on the platform's community pages. Participants were recruited through maximum‐variation and criterion‐based purposive sampling strategies (Palinkas et al., 2015; Suri, 2011). Maximum variation sampling was chosen to enhance the study's scope and represent the views of a diverse range of exercisers. In line with this approach, we did not include any exercise thresholds and allowed participants to self‐identify as ‘engaged’ in on‐demand exercise. This led to the recruitment of participants who engaged in between 3 and 10 sessions per week. Criterion‐based sampling was used to recruit participants who were aged 40–64 and who agreed with a statement adapted from exercise identity literature (Anderson & Cychosz, 1994) and group identification scales (Cruwys et al., 2020):

I consider myself an exerciser and use it when I describe myself to others. For me, being an exerciser means more than just exercising as I need to exercise to feel good about myself and feel a togetherness with other exercisers.

As this paper is part of a larger project exploring social identity processes in three online exercise platforms the term ‘exerciser’ was used for consistency across all contexts (for recruitment purposes only). Following ethical approval from the first author's institution, 20 participants were recruited (19 female and 1 male, *Mage* = 50.13 years, SD = 6.97). The opening interview question explored the context of their identification as an exerciser (e.g., *When for you, did you first start identifying with exercise as part of who you are?*) (*Myears identifying as exerciser* = 23.95 years, SD = 11.02), before exploring their participation in group exercise on demand. For context, group exercise on demand has existed for many years, first in video format, then on DVD, and now online. While the participants were recruited from a specific popular on‐demand social media community, many participants had engaged in on‐demand exercise participation (e.g., via dvd's) that predated the launch of this particular online platform (*months exercising on demand* = 37.5 months, SD = 24.18). However, for clarity, twelve participants (60%) noted that the 2020 lockdowns contributed to their introduction to group exercise on demand.

### Procedure

#### Interview one

Participants were invited to attend an online, semi‐structured interview to examine their exercise history and experiences before participating in on‐demand group exercise (e.g., *How has your identification as an exerciser changed or evolved over time?*). The interviews then explored their motivations to exercise online (e.g., *What led you to get involved in online exercise?*) alongside the role others play (e.g., *What does having other exercisers around do for you?*). The first interview ranged in length from 29 to 52 minutes (*M* = 42 minutes, *SD* = 8.66).

#### Two‐week exercise diary

After the first interview, participants were asked to fill out a post‐exercise session diary for two weeks. This two‐week timeframe was used to prevent participant interest burnout while also ensuring a variety of on‐demand sessions were captured in the diary period. Diaries were chosen for data collection to explore group identification immediately after exercise sessions. Participants were given the choice of completing diary entries after each session unprompted or using the diary prompts provided. These prompts explored potential facilitators of identification related to that on‐demand session (e.g., *today's exercise session reflects who I am because…?*). Research by Day (2016) has highlighted the challenges of using diaries related to both the data capture process (i.e., participants might struggle to recall and document experiences accurately) and the motivation to complete them (i.e., participants losing interest or failing to complete their diaries). To reduce these issues, participants could record their diaries in video or computer‐typed formats. Participants returned the diary to the first author after each entry or at the end of the two‐week period. The diaries ranged from 7 to 19 entries (*M* = 12.93) and from a total of 713 to 4327 words in length (*M* = 1855 words), creating 207 entries with 18 participants (90%) completing this stage. Five participants completed more than one entry a day for some days. Two participants who completed the first interview were sent the research diary briefing email but did not respond to follow‐up emails and were, therefore, not included in this stage of data collection.

#### Interview Two

The purpose of the second interview was to investigate themes discussed in the first interview and diaries. Before this interview, the researcher reviewed each participant's initial interview and diary entries to create a set of participant‐specific questions. This aimed to explore further what facilitated their identification, whether it changed over time, and any ambiguities in the diary entries. Each interview started with a review of their experience of completing the diary and what it might mean to them (e.g., *Did it reveal anything to you?, If someone was to have read those diaries, what story do you feel it tells?*). Then, we explored what they felt contributed to their identification (e.g., *What makes you proud about your participation in this on‐demand activity?*). Finally, questions related to their perceptions of their future participation were discussed (e.g., *Five years from now, what do you think your participation in on‐demand group exercise looks like for you?*). The second interview ranged from 27 minutes to 62 minutes (*M* = 43 minutes, *SD* = 8.88) in length, with 17 participants (85%) completing this stage. In addition to the two participants who dropped out after the first interview, a third participant withdrew despite having completed and submitted their diary to the researcher.

### Data analysis

All collected data, including those who dropped out, were analysed. Audio and diary content generated 650 pages (font size 12, single‐spaced) for analysis. An eight‐step abductive thematic analysis was employed, with steps interacting continuously rather than being strictly sequential (Thompson, 2022).

Firstly, data were transcribed, and familiarization commenced during collection to inform the questions for the second interview, aiding the researcher in engaging deeply with participants' experiences. Secondly, handwritten notes were coded using qualitative software (https://delvetool.com), creating an online database for collaborative coding and supervision. Third, a codebook was developed, assigning labels and meanings to codes, linking data, themes and theory (Thompson, 2022). Fourth, themes were developed by merging and refining related codes through iterative groupings. Fifth, thematic relationships were theoretically examined to evaluate how social identity processes explained the findings or revealed gaps. Sixth, themes were explored across contexts, considering differences in participants' use of various on‐demand formats (e.g., spinning, weights, martial arts, choreography). Seventh, draft results mapped codes to themes and theoretical insights to facilitate critical peer discussion. Finally, in stage eight, the themes were written up.

### Methodological rigour

In line with a relativist approach, rigour was enhanced through several methods. A critical friend process encouraged reflective dialogue and valuable feedback, resulting in a reassessment and refinement of initial themes (Smith & McGannon, 2018). The aim of this process was not to validate the developing themes but to prompt further consideration and discussion of the data analysis and deeper exploration of contextual and life‐stage‐specific social identification facilitators. Presenting findings at an international conference enabled external reflection on the research consistent with the relativist ontology (Wadey & Day, 2018) and enabled discussion about their generalizability (Smith, 2018) prior to publication. Conversations with delegates who had researched social identity in exercise settings, including SIMIC, affirmed the study's contribution to contextualizing identity transitions and deepening understanding of social identity in this domain (Jetten et al., 2010), indicating the findings' analytical generalizability (Smith, 2018). Self‐reflexivity and understanding of the exercise‐on‐demand community were essential throughout. The first author, a midlife adult and lifelong fitness participant, adopted a reflexive stance, supported by a reflexive diary, due to shared demographics with participants. Before data collection, immersion in the on‐demand community (e.g., sessions and Facebook groups) aided in understanding platform structures, formats and language. This experience, alongside familiarity with the online platform, supported rapport and openness consistent with prior community‐based research (e.g., Le Dantec & Fox, 2015). The aim of these steps was to recognize and gain awareness of the impact the 1st author would have on the data.

## RESULTS AND DISCUSSION

Thematic analysis of social identification in on‐demand participation revealed a shift from in‐person to online group identification, allowing participants to maintain a valued identity as exercisers. This transition is captured through three interconnected themes outlining key facilitators and processes: (1) Context of group exercise on‐demand provides midlife agency over exercise continuity; (2) content launches are the collective event that initiates the group bond and (3) challenges facilitate connection to others in midlife.

### Group exercise on‐demand provides midlife agency over exercise continuity

This theme captures participants' shift from identifying as in‐person group exercisers to embracing on‐demand group exercise. They gradually recognized both practical (e.g., time constraints) and perceptual (e.g., feeling out of place) challenges in attending in‐person classes. In contrast, on‐demand exercise offered participants a sense of agency in midlife, along with social support through Facebook communities. Prior research shows that a strong group identity encourages members to adopt sharing norms, adherence to these norms (e.g., reciprocity, privacy) and contributes to psychological safety and connection (Rathbone et al., 2023). Additionally, group belonging strengthens when members share goals and characteristics, merging individual identities into a collective one (Blanchard et al., 2023). The on‐demand format supports this sense of belonging by aligning with midlife schedules and group norms.

#### The growing inaccessibility and midlife exclusion of in‐person group exercise classes

Many participants described shifting attitudes towards in‐person classes in midlife, explaining their preference for on‐demand exercise groups. One participant expressed growing frustrations with in‐person formats: ‘when there's other people [in the gym] and you have to negotiate space. That [has] always annoyed me’ (Participant 17 – interview 1). For some, this was more than annoying, as one exasperated participant said, ‘I am just over the fighting, trying to squish 40 people into a room at 5.30 in the morning, there would literally be fights’ (Participant 3 – interview 1). Participants reported changes in their interactions during classes, noting that increased awareness of ageing and midlife led them to move away from in‐person classes. One described feeling like an outsider in midlife because they are not in the same age group, stating:

There was a lot of young people [in the exercise class] and I just wasn't finding it particularly friendly, that's all. I was probably at a stage in my life when I might've been looking for that [friendliness] a bit more than I normally was […] I just think I've got a little bit older. I've gone out of that demographic a little bit. (Participant 15 – interview 1)

The transition away from in‐person group exercise classes was often linked to comparing themselves to other participants. One participant discussed how comparing themselves and seeing others as different contributed to feeling like they didn't belong: ‘I wasn't regular, I think I had a bit of an imposter syndrome because I was always comparing myself to the other people, I did feel a bit like I didn't belong’ (Participant 16 – interview 1). Participants described a gradual, pervasive feeling of not fitting into in‐person classes that was connected to their various midlife identities. One participant explained that being a midlife parent, exerciser and someone with health challenges made in‐person classes less suitable, leading to a shift towards on‐demand exercise and a sense of no longer belonging to the in‐person ‘crowd’:

We are grown‐ups and realize that it's [exercising] not always the first priority. Especially for her [referring to her friend], her daughters are about to do her A‐levels. So, she's got a lot of other stuff going on. We've both had various health problems, which is impacting what we can do. […] I'm over 40. I know that I need resistance work. Muscle tone is not what it was when I was younger […] I have no real interest in going back to a gym. I genuinely prefer not being in crowds anyway. For me, I pretty much fully converted to the on‐demand model. (Participant 4 – interview 1).

Research shows that perceived similarity strengthens group belonging (Campbell, 1958; Easterbrook & Vignoles, 2013). Participants noted that growing awareness of reduced similarity to in‐person members led to feelings of midlife incompatibility and a sense of non‐belonging in such groups. While identity change is often linked to event‐based transitions, (e.g., retirement; Haslam et al., 2023) this evidence suggests that life‐stage transitions, such as midlife, may emerge more gradually.

Group exercise classes on demand empower midlife agency over participation and social interaction. A key theme for all participants was the empowerment that came from accessing group exercise on demand. Unlike traditional classes with fixed schedules, on‐demand options allow users to work out at any time and from anywhere, giving them agency over midlife restrictions such as limited time and control. One participant shared that this flexibility made it possible to combine midlife family time with exercise:

I could make them [on‐demand classes] fit my schedule. Online [exercise] bends time and space, you can have it wherever you want. I can take it with me when I go on holiday. I can bring the kids. I don't have to go, ‘bugger it! I've missed half of [weights class].’ I just turn it on when I want. (Participant 12 – interview 1)

Participants found that exercise participation and interaction vary between in‐person and on‐demand group formats. In in‐person classes, participation and interaction happen simultaneously, whereas in on‐demand settings, individuals exercise and socialize at their own convenience. They observed that on‐demand interactions were more support‐oriented, providing both emotional and practical help. Participant 9 (interview 1) shared:

And then I discovered that I can do this [exercising and socializing] by myself whenever I want, however long I want, as I can always fit it into my schedule. So that was the winning thing for me. I started joining the [on‐demand] groups on Facebook. I was seeking some advice on the equipment and that drew me in.

The growing agency participants associated with their on‐demand participation also extended to a re‐evaluation of the expectations of in‐group social interactions. One participant illustrated how participation in on‐demand classes removed the social pressure associated with in‐person exercise:

If you are in a [in‐person] class, sometimes the instructor expects you to applaud or connect with them because it's important for the instructor. Whereas if I am with on demand, I don't have to greet the instructor. I don't have to please the instructor. (Participant 6 – interview 2)

As an emergence‐based identity transition, midlife presents time‐based challenges that limit participation in in‐person exercise classes. These classes often lack social interaction, as participants typically arrive and leave at set times with minimal informal engagement. In contrast, on‐demand exercise offers flexibility, allowing social groups to create interaction and establish shared norms. Participants expressed complex social needs in midlife, wanting to be around others without engaging directly (i.e., social but not necessarily sociable). With on‐demand classes, participants gain agency over social interactions, which helps establish one of the in‐group norms and a sense of similarity among participants:

[when attending in‐person classes] I would normally hang back in the back. And it's not that I needed to be alone, it's that I don't necessarily wanna have to engage with a whole bunch of people […] So with an [on‐demand class], that feels like a class to me. I appreciate having others there and also experiencing it, but the thing I appreciate [with on‐demand classes] is not having to interact with anybody. (Participant 10 – interview 1)

Research shows that when a group values individualism, members may act independently but still feel they adhere to group norms and belong (Jetten et al., 2002). On‐demand exercise supports midlife needs by promoting flexibility and choice as group norms, shaping members' involvement and social interactions. Results indicate distinct deindividuation processes between in‐person and online exercise groups. On‐demand exercise replaces fixed schedules, class lengths and social pressure with flexible schedules, varied durations and minimal social expectations. This suggests that online in on‐demand groups, activity identification (e.g., participating in a class) occurs separately from intergroup interactions (e.g., chatting with others on social media). This separation offers new insights into social identification and group compatibility mechanisms in on‐demand settings (Jetten et al., 2010).

### Content launches are the collective event that initiates the group bond

A collective group event (i.e., the launch of a new programme) united participants in the discovery and experience of the new routines. Research shows that shared experiences foster group formation and social identity (Neville et al., 2022). The new programme launches acted as catalysts, inducting participants into group activities, promoting in‐group belonging and fuelling social media participants' introductions. All participation in this group exercise is anonymous, which participants said made them more willing to try new formats they might avoid in in‐person classes, as anonymity removed social judgement.

#### Content launches signal a collective event

Participants described how new ‘challenges’ (e.g., a new programme of classes suggested to be taken in a specific order) motivated them by feeling like events. One participant explained how taking part in such an ‘event’ strengthened their identification with and connection to the community:

A new challenge from [on‐demand platform] has hundreds of members all following the same programme over the next 2 weeks. This is really exciting as it makes me feel even more part of a community around fitness. This really emphasized exercise as part of my identity as I felt I was doing this with like‐minded people, even though they're people I barely know and have only got to know via the [Facebook] community. (Participant 20 – diary)

The participant diary entry above was later explored as part of their 2nd interview, where they elaborated on their experience and the impact this had, linking their perception of the group energy or ‘vibe’ with their intentions to ‘give their all’ (i.e., giving max effort) when exercising:

I think I felt motivated by that [taking part in the challenge]. I think the few days before you sense there was a bit of a vibe, a kind of effort to get going. I'm gonna give that everything because other people are doing it at the same time and there's something really driving about knowing that. (Participant 20 – interview 2)

Although they were alone and unsure if others were participating simultaneously, this participant felt that their group identity was strengthened by perceiving others as doing the same, which motivated them to expend greater effort. This reflects a self‐categorization process of in‐group prototypicality in online exercise, where perceptions—shaped without visual cues—allow participants to imagine fellow group members. Participants also described how new challenges led them to engage with dedicated Facebook communities for peer support and feedback, encouraging them to try new workouts. These social media spaces offered shared knowledge and vocabulary that signalled in‐group membership, reinforcing their sense of belonging to a broader fitness community. As one participant noted, this connection between new content, social media engagement and admiration for knowledgeable members shaped their community experience:

I think encouragement and being able to try different things, and some people will ask, ‘I really liked this [new] release. Are there any that have a similar energy?’ And some people in there, they must know them all, like inside out. (Participant 8 – interview 1)

The launch of new releases and challenges created a shared crowd experience, contributing to a sense of group identification and anticipation. Participants were driven by fear of missing out and a desire to be part of the moment. Crowd psychologists have examined how individuals and groups influence perceptions and behaviour (Drury, 2020). Research distinguishes physical from psychological crowds, with the latter defined by shared identity and coordinated behaviour (Templeton et al., 2018). This reflects the findings here, where, despite no physical or synchronous activity, participants visualized a group, enacted group identity and reinforced connections with others (Hopkins et al., 2016).

#### Exercise anonymity socially de‐risks new content trial, creating a safe and inclusive space

The content of the challenge programs exposed participants to new classes that they would not have tried without the introduction of the challenge programs, as one participant shared, ‘I really like [the challenges] because, through the challenges, I found other classes that I would not immediately go to, so I discovered the rpm and strength [classes]’ (Participant 11 – interview 1). Another participant noted that exercising anonymously encouraged them to try more classes than they would in person due to reduced self‐consciousness stating, ‘I feel a bit less self‐conscious trying at home. It's only me that can see me, tripping over trying to do the dance moves. So, I've tried a bit more variety [of exercise formats]’ (Participant 14 – interview 1).

After completing the challenge programs, participants noted that on‐demand exercise allowed for anonymity, removing the fear of judgement associated with in‐person classes. Without social pressure, they could exercise freely and set their own expectations for effort. In discussing this sense of permission, they reflected on shifting group norms, often using rhetorical questions to challenge in‐group expectations:

If I don't achieve it [the desired repetitions] or if I messed up the steps. No one knows. It doesn't matter, does it? There's only me that knows. You don't want other people to think that you couldn't do it. It shouldn't matter, should it? but in my home, no one can see. (Participant 7 – interview 1)

This participant felt the exercise content and on‐screen diversity (i.e., having different people and abilities within the same exercise class) signalled that adapting the class to their abilities was the group norm, endorsing personalization as an inclusive practice:

You can see other people on the screen, and I do quite like that. Being able to see lots of people doing it in slightly different ways. You feel like there isn't a right and a wrong way there are options. (Participant 7 – interview 2)

Although physically alone, participants felt safe and connected to the on‐screen group, empowered by their anonymity. This reflects an online version of relational vulnerability (Corlett et al., 2021), where trusted virtual environments (e.g., virtual exercise settings) enable the exercise of agency, connection and reduce the vulnerability associated with starting new routines. Engaging with instructors created a sense of shared experience and social inclusion, while anonymity reduced social judgement and offered control over participation. As illustrated by Participant 5 (interview 1):

It doesn't matter where you start [on the challenge programme], from the online perspective, it's very safe. You're watching them [the instructors], you're feeling you are in it with them, but nobody can see you. So, if you're not doing all the reps, […] if you have to pause it so you can have a drink of water and can't do some of the stuff, you're still doing more than you would be [if it were an in‐person class] and you're making progress.

Exercise anonymity enhances inclusion by reducing social risk, creating a perceived ‘safe space’ and signalling an inclusive norm where individuals can participate in their way. The lack of visibility (i.e., both being seen and seeing others) allows participants to engage in the moment without fear of judgement. While group homogeneity is typically seen as developing over time (Oakes et al., 1995), challenge programs foster immediate homogeneity by introducing new content unfamiliar to all. This shared learning becomes a group norm supported by the anonymity and autonomy of on‐demand formats. These findings challenge earlier claims that visual anonymity does not influence self‐categorization or group identification (Lea et al., 2001), instead suggesting that the absence of others' anonymity allows participants to self‐categorize and identify with the group through participation alone.

### Challenges facilitate connection with others in midlife

Despite exercising alone physically, the challenge programs connected participants with instructors, on‐screen participants and a broader community via social media. This fostered a sense of midlife connection and support through on‐demand group classes. Stevens et al. (2022a) found that perceived similarity enhances engagement and attendance, with instructors playing a key role in building group identity (Stevens et al., 2022b). Participants described forming a relationship with the on‐demand community through screens (e.g., TV for participation and the internet for social media) before, during and after sessions.

#### Midlife company and connection during classes

Participating in new challenges offered opportunities to meet diverse life‐stage relatable instructors and on‐screen participants [many classes feature actual participants]. Participants reported that on‐demand exercise reduced loneliness and promoted social interaction during midlife, supporting the idea that group participation acts as a social cure (Jetten et al., 2009) by enhancing health and resilience through connections, especially amid midlife stresses (Jetten et al., 2010). Watching others on screen gave participants a sense of company while exercising alone, valuing relatable instructors and feeling connected to them. One participant noted that familiarity and similarity enhanced their perceived relationship with instructors when they shared:

Because of the challenges, I have [instructor name] in my life three times a week, so [instructor name] is a constant presence in my life, a familiar face. I enjoy it when they throw in, a couple of older people, people I can relate to, as in a life stage kind of relationship, [another instructor] is not one of the energy bodies. She's a mature woman in her forties, like myself. I think I relate a lot less to the young ones. (Participant 16 – interview 1)

Research by Easterbrook and Vignoles (2013) found that viewing a group as a ‘social category’ and feeling similar to its members (e.g., identifying with midlife) enhanced belonging. This sense of belonging grows stronger when members view others as representative (i.e., prototypical), reinforcing their social identity (van Knippenberg et al., 2024). Similarly, Stevens, Fitzpatrick, and Cruwys (2022) found that similarity boosts engagement and attendance.

Participants discussed how relatable struggles (e.g., keeping up with the exercises) connected them to the instructors and on‐screen participants. Reflecting in her diary, Participant 16 felt a sense of relief hearing the instructor struggle, which motivated her to keep going: ‘that helped me not give up when it got tough. [instructor name] noises are also a source of relief; he is feeling it, and I find it relatable’. The shared struggle was a common theme and extended to how participants related to the other participants they could see in the filming of classes. Witnessing others struggle increased their sense of connection and reduced feelings of isolation due to perceived shared experience (i.e., struggling with the exercise), as illustrated by Participant 17 (interview 2):

You can see other people in the audience, right […] it was nice to see other people struggling the same way you felt like you were struggling. Other people like doing well, like not just the instructors, right? Like it's ‘oh, that's someone similar like me.’ So, like it didn't feel as alone. I know it sounds weird.

Research on social identity leadership shows that leaders seen as reflecting ideal group traits are more effective and trusted than those perceived as average (van Knippenberg et al., 2024). This supports Stevens, Fitzpatrick, and Cruwys (2022), who found that instructors' language and use of descriptive norms help build resilient identities, with behaviours like showing vulnerability (i.e., seeing them struggle) aiding identification and norm formation. Though physically alone in class, participants reported that engaging with on‐screen instructors and peers enhanced motivation and group identification. They often mirrored behaviours typical of in‐person interactions:

I always talk to the presenter, they're going, ‘come on, who can do this?’ You're like, ‘you know what? I'll give it a go.’ I talk to the people doing it, shouting at the screen. It really does help, here's a real sense of connecting with them. I don't know why […] ‘cause they don't know I'm there doing it. (Participant 7 – interview 1)

Participants felt a sense of belonging in the on‐demand community, viewing peers and instructors as friends. It filled a midlife social void, offering a social cure and fostering bonding through shared experiences and similarities. Shared experience is a fundamental basis for connection, belonging and support, where common circumstances create inclusion and minimize isolation (Bradshaw & Muldoon, 2020). With Participant 4 (interview 2) stating:

I guess it's [participating in on‐demand classes] being part of the community and then realizing that other people are sharing a similar experience because they're doing the same activity, you have the same friends, with the instructors, who are your friends.

Krishna and Götz (2024) demonstrated that motor coordination (i.e., performing the same action simultaneously) can induce social identity. This data suggests that combining perceived motor coordination with emotional connection via ‘through screen’ interaction (i.e., feeling the same thing simultaneously) may help bridge midlife loneliness and social interaction gaps by providing relatable new online friends. A previous social prescribing study showed that increasing social interactions enhanced community belonging, provided social support and reduced loneliness, while improving quality of life (Wakefield et al., 2022).

#### Midlife community outside of classes enhances identification and provides social support

All participants highlighted the crucial role that on‐demand social media pages played in providing a supportive post‐class community during midlife challenges. Sharing on‐demand experiences helped them express and build their sense of belonging to something special in midlife. One participant implied their shared ownership over the group using ‘we’ to signal the positive distinctiveness, emotional belonging and psychological closeness they felt through in‐group affiliation. They stated, ‘I want to think that it's almost like a secret club. we have this shared interest, and we are all over the world. It's really exciting, like a really cool thing to belong to. It's rewarding’. (Participant 16 – interview 1).

Participants saw the social media group connection as a two‐way process; it encouraged accountability, making it feel more like a group. One participant said this accountability boosted their exercise effort because, ‘without this kind of external stimuli [accountability to the group], I wouldn't push myself as much, it's more interactive. It feels like a group’ (Participant 10 – interview 2). Participants expressed a desire for accountability that extends beyond effort and adherence to exercise, highlighting a need for social support and others to care about their progress. One participant emphasized the importance of this supportive, non‐judgemental community:

And it's lovely when you look on social media and people say, ‘oh my God, I managed a full 30 minutes’, I think it's non‐judgemental because you are in control of your progress […] there's that support, that checking in, to share the progress you're making and I think that is really important. (Participant 5 – interview 2)

While acknowledging being physically alone when exercising, participants felt that participating gave them conversational social currency (i.e., context‐relevant things to chat about with other participants) and that being in those conversations motivated them:

When I exercise at home, I exercise by myself, but there is something about the energy of that [social media] group. People would say, ‘Hey, so I did this workout today’ and I'm like, ‘I should do that.’ then it allows me to comment on their post and continue that conversation. (Participant 9 – interview 2)

Participants saw the social media community as a safe space to step back from midlife demands and external expectations. One described using it to manage midlife pressures while engaging in something both meaningful and significant, highlighting the group's role as a psychological resource (Jetten et al., 2017), stating:

I really enjoy the time that I see people around all the world doing the same classes that I am doing. It makes me feel like I'm part of something greater. it's something you feel comfortable with. It's my extended family. It's a place where I can be myself, disconnect from everything else and feel good.(Participant 6 – interview 1)

Participants noted that engaging with on‐demand social media pages contributed to their midlife in‐group identity, describing a supportive, transnational ‘secret club’ where shared experiences and mutual encouragement foster emotional belonging. This reflects the ‘social cure’ literature, which suggests that identification with meaningful groups enhances well‐being by providing belonging, support and purpose (Haslam, McMahon, et al., 2018). Referring to themselves as ‘we,’ participants showed positive distinctiveness and psychological closeness; shared workouts and encouragement strengthened both group membership and personal accountability.

Our findings support Häusser et al.'s (2020) distinction between individual, group and perceived group identification. Within the on‐demand community, participants' self‐categorization as midlife exercisers (e.g., individual identification) coexisted with a sense of belonging to a supportive, transnational ‘secret club’ (e.g., group identification). Frequent use of ‘we’ indicated recognition of this bond by others (e.g., reciprocal group identification). This alignment activates mechanisms that Häusser et al. (2020) link to better health, including collective efficacy and social support at the group level, and adaptive stress appraisal and self‐attribution at the individual level. These results also reinforce the ‘social cure’ model (Haslam, Jetten, et al., 2018), showing how shared workouts and mutual encouragement build a sense of membership, accountability and psychological resources for well‐being.

## GENERAL DISCUSSION

This study explored social identity experiences associated with on‐demand group exercise during midlife. Findings revealed a gradual shift from in‐person exclusion to online inclusion, facilitating the continuity of group exercise in midlife. A key finding was that new content launches acted as collective events, galvanizing the community, encouraging engagement and inspiring format experimentation. The study contributes to understanding social identity in online exercise, showing that on‐demand platforms can offer personal agency, anonymous judgement‐free participation and a sense of social connection during and after workouts.

### Contributions to theory and research

This study makes three contributions to the social identity literature by providing the first study of social identity processes in online group exercise contexts where people cannot interact when undertaking the activity. Firstly, this study adds to the nascent research into social identity within online exercise groups by highlighting the difference in the facilitators of social identification related to different contexts. In the one study that has explicitly explored facilitators of social identity in online exercise, Richards et al. (2025) found that Zwift riders recognized their similarities with others based on age, as all riders' ages are publicly visible in on‐screen profiles on the platform, which contributed to their sense of identification. By comparison, in this study, similarity was perceived, as anonymity in on‐demand platforms removes the opportunity for factual comparison and live interaction with others. Participants in this study emphasized that their perceived life‐stage similarity with other participants was associated with shared life‐stage demands, and that in‐group belonging was based on the assumption and desire for asynchronous, invisible and anonymous participation.

Having the ability to participate as a group member anytime, anywhere—with full control over which classes to attend—and anonymously offered greater inclusion by increasing group exercise availability and removing social judgement around participation. This provided an opportunity to sustain exercise activities that support identity continuity and aligned with their life‐stage demands. Specifically, participants highlighted how their midlife desire for control over exercise (e.g., timing, location and type) and post‐exercise social interactions (e.g., chatting on dedicated social media pages) were similar. Thus, these findings suggest that in on‐demand exercise contexts where people cannot interact in real time, participants experience identification with a perceived in‐group, which simultaneously helps them feel group entitativity and connectedness to others in their heads when exercising (Brown & Pehrson, 2019). This also fosters feelings of support and allows individuals to be supportive of others via social media when not exercising, despite being physically alone.

Secondly, this research highlights broader implications for social identity research within online groups. Evidence from this study suggests that online, on‐demand groups can offer an at‐home and anonymous route to group membership. The findings from this research reinforce Jetten et al. (2002)‘s idea that individuation can become a group norm when being individualistic is seen and maintained as the primary norm. For instance, participants in this study perceived that the group valued participating in the group activity (i.e., exercising) at their own pace and anonymously. Rather than group identity being strengthened via anonymity concealing individual traits as suggested by Postmes et al.'s (2001) research, this study suggests that anonymity, when combined with asynchronous participation, strengthens identification through enabling individual agency (i.e., empowering individual traits).

Previous research indicates that when people experience uncertainty, they are especially likely to seek group identification and a sense of shared reality (Hogg & Rinella, 2018). Extending Cheng and Guo's (2015) suggestions that interaction and knowledge exchange contribute to social identification and group membership, this study suggests that the uncertainty that comes with learning new content creates opportunities for discussion and sharing on social media. Regularly released content gives participants a shared learning experience, encouraging them to interact online (e.g., by exchanging tips, discussing their progress) and offering each other social support. The new content levels the playing field by establishing ‘continuous learning’ as a galvanizing group norm. This prevents the development of an in‐group knowledge hierarchy because everyone is learning the new content at the same time, creating a more inclusive environment where everyone continues learning at the same pace. This suggests that this shared experience (i.e., learning together) and perceived similarity (i.e., in terms of learning stage) increase identification.

Thirdly, this study contributes to our understanding of identity transitions that could extend SIMIC. Specifically, the findings suggest that emerging transitions (e.g., ageing) differ from event‐based transitions—where an explicit change ‘event’ marks a clear point of identity transition (e.g., retirement; C. Haslam, McAulay, et al., 2024; Pachana et al., 2017)—as they involve subtle, incremental shifts that are often hard to detect and address. By comparison, this study suggests that emergent‐based identity transitions are initiated by self‐evaluative recategorization processes that involve reflection and comparison, previously highlighted by Tesser (1988). As midlife adults assess their compatibility with existing groups (e.g., as a member of an in‐person group) via social comparison and context evaluation, they begin to review the multiple points to which their identity is moored. While existing SIMIC research highlights remooring processes that happen after an identity transition (i.e., attachment to a new identity related to a previous one) (Zhang et al., 2025), this study highlights a process that happens before transition. These findings reveal the impact of this self‐evaluation recategorization process specific to emergence‐based identity transitions.

The self‐evaluation process that results in recategorization leads people to demoor and deconstruct their identification with that group (i.e., detach from a previous identity). This deconstruction happens in two parallel processes (i.e., deductive social identity deconstruction and inductive social identity deconstruction). In terms of deductive social identity deconstruction, people deconstruct their identity when they no longer *see* themselves as prototypical members of the group compared to other in‐group members. As illustrated by Participant 16 who described demooring from in‐person group exercise when feeling imposter syndrome after comparing themselves to other regular and typically younger in‐person exercise group members. Additionally, inductive social identity deconstruction occurs when people are no longer able to act like group members in exercise contexts because of personal circumstances (e.g., midlife challenges and demands) that mean they are unable to align with group norms (e.g., attend and engage in in‐person classes). This was seen, for example, in the case of Participant 4 who discussed how the combination of prioritizing midlife responsibilities (e.g., parenting) and the need for age‐specific exercise (e.g., midlife specific strength training) led to them demooring their exercise identity from in‐person group exercise. Subsequently, participants in this study remoored their exercise identity through on‐demand exercise as it met their midlife needs which allowed them to maintain identity continuity via a new online group membership (Zhang et al., 2025).

### Applied implications

These findings suggest four ways online platforms can enhance social identification in exercise communities. First, group onboarding through shared events, such as new class launches, may create unity through collective content discovery. Second, Thedinga et al. (2021) found that some individuals use self‐exclusion from exercise to manage weight stigma. On‐demand platforms may engage these users by addressing exclusion factors such as culture, ability or access. Third, combining user agency with the anonymity of online participation can reduce fear of judgement, which influences participation (Seal et al., 2022). On‐demand options allow users to explore activities privately, possibly increasing identification with new groups or classes. Finally, findings support social prescribing of online exercise for midlife adults. According to Haslam, Haslam, et al. (2024), such interventions are most effective when aligned with identity‐based infrastructures and supportive leadership. Online groups can thus provide key psychological resources (e.g., connection, support, meaning, control and efficacy) identified in the social identity approach to health (Haslam, Jetten, et al., 2018).

### Limitations and future directions

While this study has several strengths, including the interview‐diary‐interview method that enabled insight from both in‐the‐moment perspectives and reflective insights over time, certain limitations suggest avenues for future research. First, as the study focused solely on current participants of one on‐demand platform, it overlooked the perspectives of individuals who had tried the platform but subsequently discontinued their use. Second, the group exercise on‐demand category has many different branded competitors. Future researchers could examine the identification processes in other on‐demand branded group exercise services to explore which insights from this study may be context‐specific (i.e., on‐demand) and which might be platform or brand‐specific. Further, participants needed the physical space to exercise, the technical know‐how to set it up, the subscription costs, devices to watch it on, an internet connection and exercise equipment. These factors can form a variety of potential barriers to participation, which may be limiting for many disadvantaged groups. To that end, a further limitation of this study comes from the core of the paper's findings namely, that the participants' identification and belonging were based on their midlife similarities, implying that representation played a key role in their relationship with the group. Future researchers could consider the role of representation of other identifying classifications relevant to the midlife life stage (i.e., single by choice, carer, divorcee, bereaved, living alone, single‐parent, child‐free, empty‐nester). Finally, although not a main focus of this study, the findings in this study suggest that anonymous, on‐demand exercise contexts may support aspects of well‐being. Building on this, future research could more directly examine how online exercise groups contribute to mental health and well‐being among adults in midlife.

## CONCLUSION

In summary, our research indicates that changes in social identity related to group exercise participation during midlife evolve and are negotiated gradually. On‐demand platforms can offer exercise continuity and agency that address many midlife challenges with in‐person attendance, such as rigid schedules and reduced similarity to other in‐person participants. Collective new exercise class events create inclusive moments where participants are ‘inducted’ into the group through mutual discovery and class participation, while maintaining anonymity to explore new activities without social pressure. Feeling supported by on‐screen instructors and online communities despite being physically alone enabled participants to be anonymous yet together.

## AUTHOR CONTRIBUTIONS

**Toby Richards:** Writing – original draft; writing – review and editing; conceptualization; project administration; formal analysis; data curation; investigation; methodology. **Matthew J. Easterbrook:** Writing – review and editing; supervision; conceptualization; methodology. **Matthew J. Slater:** Supervision; methodology; conceptualization; writing – review and editing. **Melissa Day:** Methodology; data curation; supervision; conceptualization; writing – review and editing. **Sean G. Figgins:** Writing – review and editing; supervision; methodology; conceptualization; formal analysis.

## Supporting information


Data S1:

## Data Availability

For ethical privacy reasons, the anonymized transcript data are not publicly available but are securely stored and available upon reasonable request from the corresponding author.
