# Supplementary material for: How on‐demand agency of anonymous group exercise membership supports emergence‐based social identity transition in mid‐life
Source: Br J Soc Psychol. 2025 Nov 14;65(1):e70022. doi: 10.1111/bjso.70022 (PMC12617392; doi:10.1111/bjso.70022)
Supplement: Supplementary file 1 — Data S1: [file BJSO-65-0-s001.docx]

**Supplementary File Interview 1, diary prompts and Interview 2**

Interview 1 Questions

When for you did you start identifying with exercise as part of who you are?

...How did it all begin? …What was the journey?

…How has your identification as an exerciser changed or evolved over time?

…what and who influenced you back then?

Do you prefer group exercise formats?

…What does having other exercisers around do for you?

What lead you to get involved in online exercise?

…How did you discover it?

…How did you go about buying it?

…Did you do a trial?

How did you know (platform) was the one for you?

…What other platforms have you used or looked at?

…Why did they not work for you?

…Why do you prefer - on-demand/live/ community?

…Have you tried - on-demand/live/ community?

If no…If you were going to make photo diary of your participation what would you capture and why?

…What do you capture - badges, moments, set up?

Do you have a dedicated space to exercise in?

…How would you describe your set up?

…How does your set up reflect who you are as an exerciser?

…If no…If you could have the ultimate set up what would it be?

Outside of participation in the classes, how else do you do engage or follow other exercisers/ community/ instructors?

What else do you read or watch to stay informed about ………?

…Do you follow any specific groups or individuals…..if so why

What (online platform) social media posts most stand out to you?

…What social media posts do you most relate to….and why?

…What influences that -where do you get those ideas from?

What do you post on (participation-specific) social?

…and when …

…what do you comment on?

…what inspires that?

What do you think and feel when you see participants sharing their

…photos

…activities

…set up

…results

What do you most look forward to?

What do you focus on during a session?

…What do you focus on …when it gets tough/hard?

How do you interact with others during a session?

…When do you tend to interact?

…Why do you?

…How does that make you feel?

Do you feel you are exercising alone or with others?

…What kind of connection to

…the instructors do youhave?

In what way does participating in ……. reflect who you are?

What would you miss if there was no more……..?

How would you describe the ultimate session?

…What would make that happen?

Five years from now, what do you think ……..looks like for you?

If your participation in (Group Exercise Named activity) was a story what would be the genre and plot and who are the key characters?

What do you think and feel about fellow ……. ers?

What do you think about people who don’t do …………

Is there anything we haven’t discussed that you think could be of

relevance to this study?

Diary Instruction and prompts

Exercise Diary Information:

IDEALLY AS SOON AS POSSIBLE AFTER EACH EXERCISE SESSION for 2 weeks:

Complete a diary entry in any method that suits you as if you were recording the story of that exercise session for you to read or listen back to in the future.

This can be on an email, handwritten, as a whatsapp voice recording, or as a whatsapp text message.

 Here are a range of prompts that may help you get started, but what to write is entirely your choice.

Dear diary, .

Taking part in today’s exercise session reflects who I am because…

The key moments of this exercise session were…………. because….

For me, taking part in today’s exercise session means….

People who do these exercise sessions are…

To feel, even more me, in future I will…

You can send the diary to me after each entry or wait until you have 2 weeks, whatever you’d prefer.

You can send them to this email or via whatsapp to my mobile 00xx xxxx xxxxxx

Please let me know if you have any questions.

Interview 2 – post diary and interview 1

Thank you for completing the research diaries over the last few weeks. We will chat through some of the topics you kindly shared in those today.

But before we go into today’s interview topics…

How did you find that process?

Did it reveal anything to you?

If someone was to have read those diaries what kind of story do you feel it tells?

Or what picture to you feel It would paint?

What makes you proud about your participation in ……

How do you monitor your progression on x?

What do you compare that to?

… your results

…others

Then, follow up with questions specific to the participant based on interview 1 and the diary content.
